# Gut Microbiota Response in Meagre (*Argyrosomus regius*) Subjected to a Plant-Based Nutritional Challenge

**DOI:** 10.3390/ani16030407

**Published:** 2026-01-28

**Authors:** Joana Oliveira, Marisa Barata, Rafaela Santos, Cláudia Serra, Florbela Soares, Pedro Pousão-Ferreira, Aires Oliva-Teles, Ana Couto

**Affiliations:** 1 FCUP—Department of Biology, Faculty of Sciences, University of Porto, 4169-007 Porto, Portugal; up201708342@edu.fc.up.pt (J.O.);; 2 CIIMAR—Interdisciplinary Centre of Marine and Environmental Research, University of Porto, 4050-208 Matosinhos, Portugal; 3 IPMA—EPPO—Portuguese Institute for Sea and Atmosphere—Aquaculture Research Station, 8700-194 Olhão, Portugal; 4 S2AQUA—Collaborative Laboratory, Association for a Sustainable and Smart Aquaculture, 8700-194 Olhão, Portugal

**Keywords:** biomarkers, low-abundance taxa, next-generation sequencing, low fishmeal, low fish oil

## Abstract

The gut plays a central role in fish nutrition, immunity, and overall health, making it key in aquaculture research. The microbiota, crucial to gut function, is increasingly studied as an indicator of health and nutritional status. This study characterized the gut microbiota of juvenile meagre (*Argyrosomus regius*) (initial weight 4.6 ± 0.4 g) fed for seven weeks on diets in which fishmeal (FM) and fish oil (FO) were replaced by increased proportions of plant-based ingredients, with the aim of identifying microbial profiles associated with nutritional challenge. Fish were fed a FM/FO control diet (CTRL; 55.1% FM, 11.3% FO), a low FM/FO diet (CD; 15% FM, 7% FO), or a very low FM/FO diet (ED; 5% FM, 5% FO). Next-generation sequencing analysis of gut mucosa and digesta revealed no significant differences in alpha or beta diversity among different dietary groups. *Firmicutes* dominated all samples, particularly *Bacilli*, *Mycoplasmatales*, *Mycoplasmataceae*, and *Mycoplasma*. Significant differences were only observed in low-abundance taxa (<1%), with higher abundance of *Thermoactinomycetales* (*p* = 7.71 × 10^−4^), *Thermoactinomycetaceae* (*p* = 7.71 × 10^−4^), *Kroppenstedtia* (*p* = 1.70 × 10^−3^), and *Pseudogracilibacillus* (*p* = 0.039) in challenged groups. This study highlights the potential of low-abundance microbial groups as targets to establish gut health biomarkers in fish.

## Simple Summary

Gut microbiota has a major influence on fish health, affecting both the immunological and nutritional status of farmed species. In this study, the objective was to examine how the gut microbiota of meagre (*Argyrosomus regius*) responds to diets containing low to very low levels of fishmeal and fish oil, replaced with plant-based ingredients, and to then use this information to help identify potential biomarkers of the fish’s nutritional status. To achieve this, fish were fed three different diets: a traditional control diet, a low fishmeal/fish oil diet, and a very low fishmeal/fish oil diet. Both gut contents and gut lining were then analyzed using next-generation sequencing. Results showed that the main bacterial groups did not change with diet and that the microbiota of meagre was consistently dominated by *Firmicutes*. However, several low-abundance taxa increased in the fish that were fed the more challenging diets, suggesting that these taxa may be sensitive indicators of nutritional stress. Overall, these findings suggest that low-abundance bacteria could serve as useful markers of gut health in meagre that may support future research in aquaculture nutrition.

## 1. Introduction

Global aquaculture production is expanding to keep pace with the growing population, and with this growth comes an increasing need to enhance production and ensure it remains economically viable and sustainable. One of the strategies to do this is by focusing on improving the feeds used in aquaculture, as they have a significant impact on fish health and growth, and are also one of the main production costs [1,2]. Over the last few years, dietary fish ingredients have been reduced and replaced by more sustainable alternative ingredients, namely plant ingredients [3,4,5]. Plant ingredients can, however, negatively impact fish health, particularly in carnivorous fish [6]. This adverse effect is not always rapidly detectable from a zootechnical or even physiological standpoint, and chronic malnutrition effects can pass undetected for extended periods [7].

The fish gut plays a crucial role in the overall nutritional, immune, and welfare status of fish and has been the focus of many studies [8,9]. Microbiota is an important component of the gut and is also involved in health status by modulating gut function, affecting digestion and absorption of nutrients, and immune status [10,11]. Imbalances in gut microbiota can lead to disease and compromise growth [12,13,14].

Feeds can modulate the gut microbiota, with both beneficial and adverse effects. For example, antinutritional factors present in plant ingredients can affect nutrient digestibility and gut morphology, leading to alterations in gut microbiota abundance and richness and a reduction in commensal microbiota [10,12,15,16]. On the other hand, functional ingredients can positively modulate the gut microbiota and reduce the adverse effects of alternative ingredients on the fish gut [17]. Therefore, understanding how feeds modulate gut microbiota can contribute to assessing the long-term effects of diets on fish health [18,19,20].

Meagre (*Argyrosomus regius*) is a carnivorous species predominantly cultivated in the Mediterranean region, and its production has increased significantly over the last decade [21], with Egypt, Türkiye, Spain, and Greece as the primary producers [22]. Despite its importance in aquaculture, information on the gut microbiota of meagre remains limited. However, analyses of the gut microbiota of meagre fed diets containing black soldier fly, using Denaturing Gradient Gel Electrophoresis (DGGE), revealed that the dominant autochthonous bacteria were closely related to the genera *Pseudomonas*, *Flavobacterium, Bacillus*, *Burkholderia*, and *Methylobacterium* [23].

NGS (Next-generation sequencing) is more powerful than DGGE as a tool for characterizing microbial communities and has contributed significantly to the study of gut microbiota [24]. NGS microbiota profiling has been applied to a variety of aquaculture species, such as European sea bass (*Dicentrarchus labrax*) [25,26], gilthead seabream (*Sparus aurata*) [12], Atlantic salmon (*Salmo salar*) [27,28] and rainbow trout (*Onchorynchus myskiss*) [29]; however, this is the first study addressing this subject in meagre. This study explores possible alterations in the gut microbiota of meagre under the nutritionally challenged conditions imposed by diets formulated to contain high proportions of plant-based ingredients, with the aim of finding potential biomarkers for evaluating nutritional condition.

## 2. Materials and Methods

### 2.1. Growth Trial

The growth trial was performed at the Portuguese Institute for Sea and Atmosphere—Olhão Pilot Fish Farming Station facilities (IPMA-EPPO, Olhão, Portugal) with meagre (*Argyrosomus regius*) juveniles at an initial weight of 4.6 ± 0.4 g. A fishmeal (FM) and fish oil (FO) based diet (55.1% FM, 11.3% FO) was used as control (CTRL), and two other diets were formulated, reducing fisheries-derived ingredients and increasing amounts of plant feedstuffs and oil—a challenge diet (CD; 15% FM, 7% FO) and an extreme challenge diet (ED; 5% FM, 5% FO). Diets were formulated to be isoproteic (48%) and isolipidic (18%), with no statistically significant differences in crude protein and lipid content. Proximate analysis and ingredient composition of the diets are presented in Table 1. Chemical composition of the experimental diets was determined using standard analytical procedures as described in [30]. Briefly, dry matter, ash, protein, and lipid contents were measured by oven drying, muffle furnace incineration, Kjeldahl nitrogen analysis, and petroleum ether extraction, respectively. Gross energy content was assessed by adiabatic bomb calorimetry.

The growth trial details have been previously provided in detail at [30]. In brief, before the growth trial, fish were submitted to an acclimatization period of 2 weeks and fed with a commercial diet (Neo Gold Blue seabream/seabass diet (48% protein and 17% lipids; Sorgal, S.A., Aveiro, Portugal)). The growth trial was conducted over 7 weeks using nine 200 L fibreglass tanks supplied with a continuous flow of filtered seawater. Nine groups of 60 fish were weighed in bulk and were randomly assigned to triplicate tanks per diet. Fish were hand-fed six times daily to apparent satiation. Water quality was maintained under stable conditions, with an average temperature of 23.6 ± 0.4 °C, salinity of 36 ± 1 g L^−1^, and dissolved oxygen near saturation (5.7 ± 0.4 mg L^−1^).

### 2.2. Sampling

After the growth trial was completed, 18 fish from each tank were sampled 4 h after the first meal to ensure that the intestines were full at the sampling time. Fish were lightly anesthetized using 0.3 mL/L of ethylene glycol monophenyl ether and euthanized by decapitation. Intestinal samples consisting of mucosa and digesta were collected under aseptic conditions from the anterior and intermediate sections of the intestine and pooled for analysis. To obtain sufficient biological material for gut microbiota analyses, fish were pooled in groups of three individuals, resulting in six pooled samples per tank. Each pooled sample was treated as a single experimental unit for downstream analyses. Samples were immediately frozen in liquid nitrogen and stored at −80 °C until further analysis.

### 2.3. DNA Extraction and Analysis

The DNA was extracted from intestinal samples using the Qiagen DNeasy PowerSoil Pro Kit (Qiagen GmbH, Hilden, Germany), following the manufacturer’s protocol. The taxonomic diversity of gut microbiota was comprehensively assessed by next-generation sequencing (NGS), targeting the V3-V4 hypervariable region of the 16S rRNA. Fifty-four samples (6 per tank, 18 per treatment) were sequenced, and libraries were generated using TruSeq^®^ DNA PCR-Free Sample Preparation Kit (Illumina, San Diego, CA, USA). The library quality was assessed using a Qubit@ 2.0 Fluorometer (Thermo Scientific, Waltham, MA, USA) and Agilent Bioanalyzer 2100 system (Agilent, Santa Clara, CA, USA), sequenced on an Illumina NovaSeq platform (Illumina, San Diego, CA, USA), and 250 bp paired-end reads were generated.

Paired-end reads were appointed to samples based on their unique barcode and truncated by cutting off the paired-end barcode and primer sequence. Paired-end reads were merged using FLASH (V1.2.7, http://ccb.jhu.edu/software/FLASH/, accessed on 1 January 2025) [31]. Quality filtering on the raw tags was performed to acquire high-quality clean tags [32] according to the QIIME (V1.9. 1, http://qiime.org/scripts/split_libraries_fastq.html, accessed on 1 January 2025) [33] quality-controlled process. The tags were matched to the reference database (Silva database, https://www.arb-silva.de/, accessed on 1 January 2025) using vsearch (V2.26.0, https://github.com/torognes/vsearch/, accessed on 1 January 2025) [34] to identify chimera sequences, which were eliminated [35] and the effective tags obtained. Sequence analyses were performed using the Uparse software (Uparse v7.0.1001, http://drive5.com/uparse/, accessed on 1 January 2025) [36]. Sequences with ≥97% similarity were assigned to the same OTUs. Representative sequences of each OTU were screened for further annotation, and the Silva Database (http://www.arb-silva.de/, accessed on 1 January 2025) [37] was used based on the Mothur algorithm to annotate taxonomic information.

Amplicon sequence variants (ASVs) identified as chloroplasts were considered dietary plant contaminants and removed from the analysis. ASVs identified as mitochondria were also removed. Additionally, ASVs labelled as “Unassigned”, identified only at the Kingdom level, and classified within the Kingdom “Archaea” were excluded before further analysis. Further filtering was conducted for the statistical analysis of relative abundance between groups, removing taxa not identified up to the genus level.

### 2.4. Statistical Analysis

Data are presented as mean ± standard deviation. All statistical analyses were conducted using pooled samples as experimental units. Alpha diversity indices and beta diversity were calculated using R Studio (version 2024.09.0;, Posit PBC, Boston, MA, USA) with the vegan package [38]. The Alpha diversity indices followed the following equations: Chao1 species richness: S_Chao1_ = S_obs_ + n_1_^2^/2n_2_, where S_obs_ is the observed number of species and n_1_ singletons, and n_2_ doubletons; Shannon’s diversity index: H’ = −Σ(P_i_(lnP_i_)), whereas P_i_ is the number of individuals of the *i*th species; Simpson’s Evenness Index: E = (1/ΣPi^2^)/S, where S is the number of species. Alpha diversity statistical analysis was performed by non-parametric Kruskal–Wallis test using the SPSS 27 software package for Windows (IBM SPSS Statistics, New York, NY, USA) and relative abundance between groups was analyzed with Kruskal–Wallis H-test using STAMP v2.1.3 software. The Tukey–Kramer multiple range test with no correction was used to determine significant differences among groups. A probability level of 0.05 was used to reject the null hypothesis.

## 3. Results

Growth performance was analyzed in a previous study [30] and is presented in Table S1. Briefly, weight gain, final weight, feed efficiency, and daily growth index decreased significantly with increasing dietary challenge, with the lowest values observed in the ED group, followed by the CD and CTRL groups. In contrast, feed intake was highest in the ED group, followed by the CD and CTRL groups.

After quality filtering, 2,874,218 high-quality sequences were retained, averaging 54,230 sequences per sample (Table S2). These sequences were clustered into 11,581 valid ASVs (Table S3). Taxa showing a mean proportion of 1% or higher within any experimental feeding condition were considered the most abundant. After filtering and rarefaction, 2569 ASVs (Table S4) were retained. Following further filtering, 398 ASVs (Table S5) remained for the analysis of relative abundance between groups.

**Figure 1:**
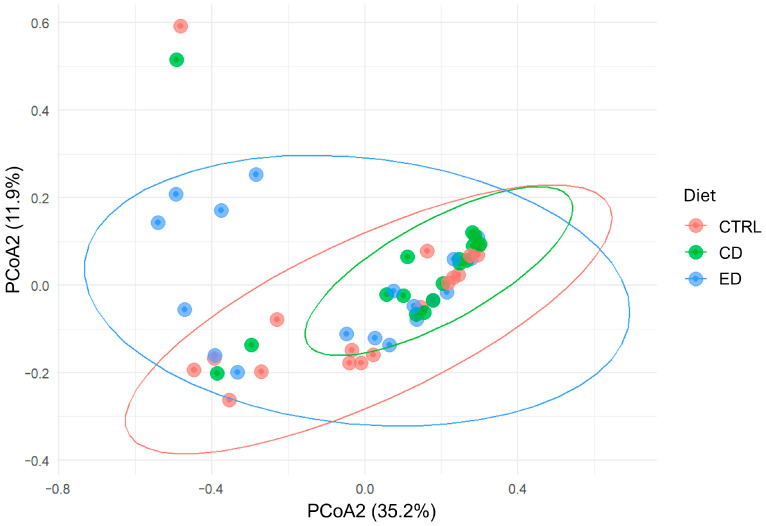
Principal Coordinates Analysis (PCoA) of Bray–Curtis dissimilarities illustrating the variation in gut microbiota composition among treatments. Points correspond to individual samples, and ellipses indicate the 95% confidence interval for each group. Treatments showed overlapping community structures, consistent with the PERMANOVA result (R^2^ = 0.057, *p* = 0.086). CTRL (Control Diet), CD (Challenge diet), and ED (Extreme challenge diet).

No significant differences in alpha diversity were observed among groups, specifically in microbial richness, diversity, and evenness indices (Table 2). Beta diversity analysis based on Bray–Curtis dissimilarities revealed no significant differences in gut microbial community composition among treatments (PERMANOVA, R^2^ = 0.057, F = 1.51, *p* = 0.097; Figure 1).

**Figure 2:**
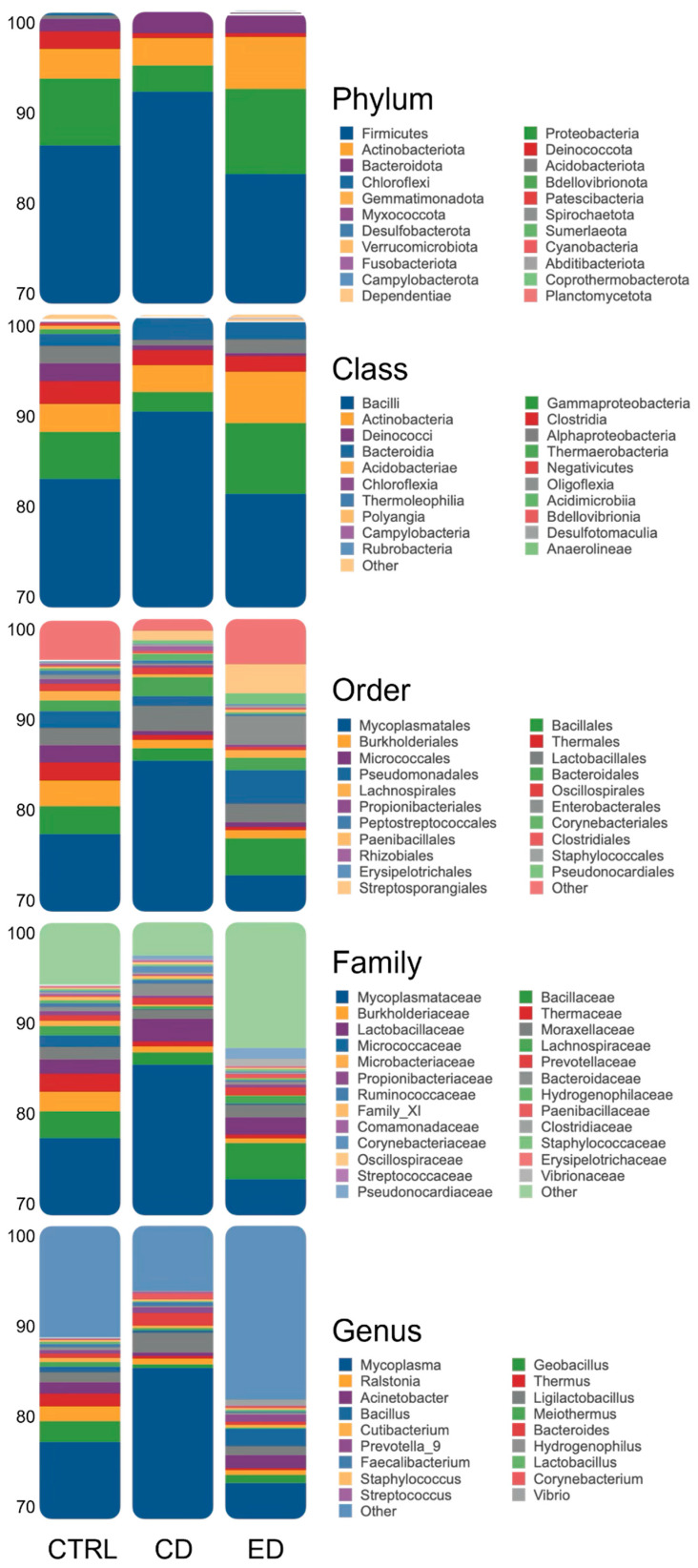
Microbial relative abundance at the Phylum, Class, Order, Family, and Genus levels in the intestine of meagre (*Argyrosomus regius*) fed the experimental diets. All Phyla are represented, while only Classes, Orders, Families, and Genera with relative abundances above 0.05% across treatments are included. Taxa not meeting these criteria are grouped under “Other”. CTRL (Control diet), CD (Challenge diet), ED (Extreme challenge diet).

*Firmicutes* emerged as the most dominant phylum across all treatments, accounting for 87% of the sequencing reads (Figure 2, Table S6). Other representative phyla were *Proteobacteria* (6%), *Actinobacteria* (4%), and *Bacteroidota* (2%), while the remaining phyla were present below 1%. At the class level, *Bacilli* consistently dominated, averaging 85%, followed by *Gammaproteobacteria* (5%), *Actinobacteria* (4%), and *Clostridia* (2%). The predominant order was *Mycoplasmatales*, with an average of 79%, followed by *Bacillales* (3%), *Lactobacillales* (2%), and *Pseudomonadales* (2%). At the family level, *Mycoplasmataceae* was the most abundant, averaging 79%, followed by *Bacillaceae* (3%), *Lactobacillaceae* (2%), and *Moraxellaceae* (1%). *Mycoplasma* was the predominant genus, averaging 79%, followed by *Ligilactobacillus* (1%) and *Geobacillus* (1%).

**Figure 3:**
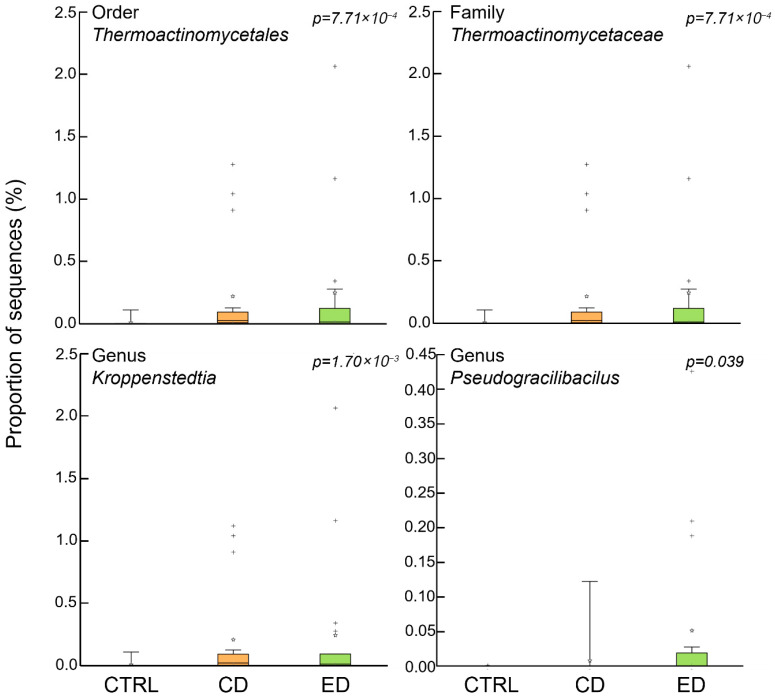
Relative proportion of sequences (y-axis) in gut samples of meagre (*Argyrosomus regius*) fed the experimental diets. CTRL (Control diet), CD (Challenge diet), ED (Extreme challenge diet). Plus signs and stars indicate outliers beyond the whiskers.

Differences in the relative abundance of taxa between groups are presented in Figure 3. Incorporating plant-based ingredients into the diets affected the relative abundance of low-abundance taxa (<1% relative abundance). Notable differences were observed in the relative abundance of the Order *Thermoactinomycetales* (*p* < 0.01), the Family *Thermoactinomycetaceae* (*p* < 0.01), the Genus *Kroppesntedtia* (*p* < 0.01), and the Genus *Pseudogracilibacilus* (*p* = 0.039).

**Figure 4:**
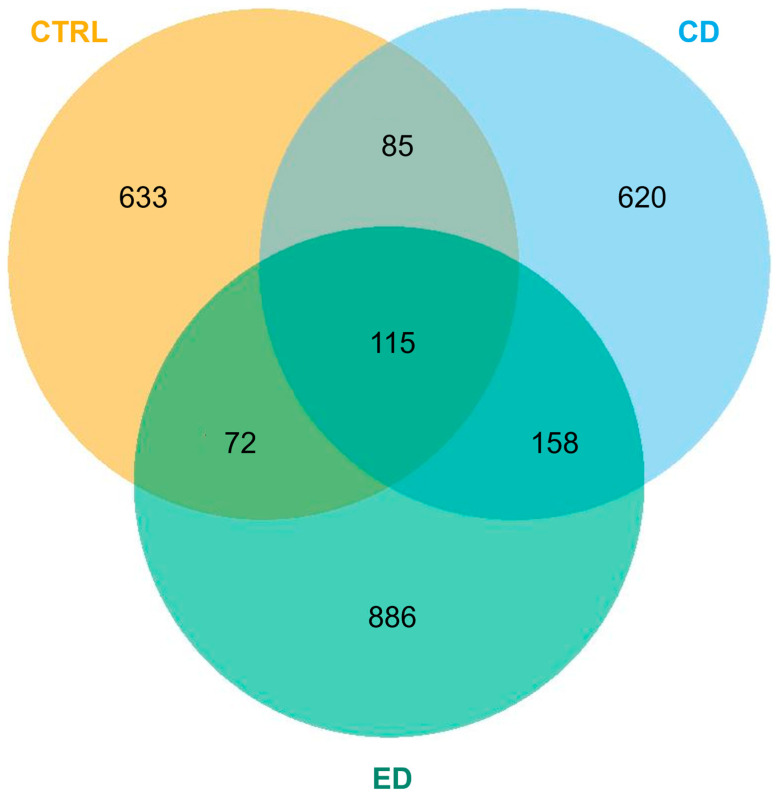
Venn diagram representation of shared and unique genera in meagre (*Argyrosomus regius*) microbiota across the experimental feeding groups. CTRL (Control diet), CD (Challenge diet), and ED (Extreme challenge diet).

As shown in the Venn diagram of Figure 4, only 115 ASVs were common to all groups, while 633, 620, and 886 ASVs were unique to the CTRL, CD, and ED groups, respectively.

## 4. Discussion

Throughout the last decade, there has been an increase in the incorporation of plant feedstuffs and vegetable oils into aquafeeds, particularly in widely produced carnivorous fish species [39]. These feedstuffs are absent from carnivorous fish’s natural diets and, even without affecting zootechnical performance, can impact gastrointestinal and overall health of fish due to the presence of antinutritional factors [40]. Therefore, detecting sub-optimal health status due to poor nutrition in fish is becoming increasingly important to avoid chronic health and welfare problems.

With the dietary inclusion of plant-based ingredients, there are typically alterations in microbiota richness, leading to either an increase [41] or a decrease in richness [25]. In the current study, alpha diversity and beta diversity showed no significant differences between dietary groups despite the pronounced differences in diet composition and growth performance, suggesting a stable core microbiota. Similar results were observed in rainbow trout fed with grain-based diets [42], where no impact was observed in microbiota despite a decrease in growth and welfare.

The most abundant phyla present in meagre gut were *Firmicutes*, *Proteobacteria,* and *Actinobacteria*, which seemed to follow the pattern observed in other carnivorous species [10,25,43]. In carnivorous fish, such as largemouth bass (*Micropterus salmoides*), rainbow trout, and Atlantic salmon, the tendency seems to be an increase in *Firmicutes* and a decrease in *Proteobacteria* upon dietary fishmeal replacement with plant-based ingredients [29,41,44,45]. However, in gilthead seabream, fish fed a high plant feedstuff diet had a higher percentage of *Proteobacteria* and a lower percentage of *Firmicutes* [12]. This is similar to what was observed in crucian carp (*Carassius carassius*)—an herbivorous species. When dietary soybean meal was increased, proportions of *Firmicutes* decreased and *Proteobacteria* increased, contrasting with findings from other studies [46]. An increase in Proteobacteria could be associated with genera that degrade cellulose [12].

In this study, despite the extreme dietary conditions, no significant differences were detected in relative abundance at the phylum or class level. The reduced differences found in this study and the mentioned discrepancy of results among other studies can be attributed to the various factors influencing gut microbiota such as environmental conditions, fish species, growth stage and gut morphology [47]. *Mycoplasma* was the predominant genus found in meagre across all treatments. Although not always found in carnivore species [48]. *Mycoplasma* was reported to be dominant in Atlantic salmon’s gut microbiota [27] and was found in high abundance on largemouth bass fed with fermented soybean meal [41]. *Mycoplasma* was suggested to play an important role in supporting gut health in Atlantic salmon [49]. In this species, low microbial variation across individuals exposed to different environmental conditions has been observed, with a high relative abundance of *Mycoplasma* present [50]. Similarly, here in meagre, strong host selection pressure may have contributed to the observed dominance of *Mycoplasma*, thereby reducing variability between fish exposed to different dietary conditions.

Various factors beyond feed can modulate the microbiota of fish. Environmental conditions such as water temperature and salinity have been shown to play an important role in the gut microbiota of fish [51,52,53]. The modulation of the microbiota by the nutritional conditions could have resulted in the specific groups highlighted, but environmental influence, although untested, cannot be disregarded.

The taxonomic groups that showed differences in abundance in fish fed the challenging diets were different from those identified in other studies on carnivorous species fed plant-based ingredients. The *Thermoactinomycetaceae* family belongs to the *Bacillales* order, and is composed of Gram-positive bacteria that form endospores and mycelia and are typically isolated from various environmental samples (e.g., soil, marine sediments) [54]. Members of this family have been studied due to having promising functional properties, namely in the production of exoenzymes and bioactive compounds [55]. However, few studies on fish have found members of this family. In Nile tilapia (*Oreochromis niloticus*), feeding with Lactogen 13, a *Lactobacillus rhamnosus* based probiotic, resulted in a significant increase in the relative abundance of an unknown genus from the *Thermoactinomycetaceae* family [56]. In Atlantic salmon, members of this family were associated with butyrate-producing bacteria, a short-chain fatty acid related to an improved immune response [57]. *Kroppenstedtia* is a genus within the *Thermoactinomycetaceae* family and its relative abundance was also shown to increase in juvenile largemouth bass fed yeast culture-supplemented feed; however, not much is known on the biological functions of this genus [58].

*Pseudogracilibacilus* is a genus from the *Bacillaceae* family and was also present in higher abundance on challenged fish. This genus is often associated with the microbiota of black soldier fly, *Hermetia illucens* [59,60,61]. Inclusively, in Atlantic salmon, members of this genus were found in the gut of fish being fed the black soldier fly meal [62]. However, there is no information of its association with plant-based nutrition.

In this study, all taxa found to be differentially abundant amongst groups have a low abundance (<1%). These low-abundant microbes should not, however, be overlooked as they may be essential to maintaining health. Studies in humans and other animals have alerted the need to dedicate more attention to these low abundant species in microbiota to fully comprehend host–microbe interactions upon various nutritional scenarios [63]. Low-abundance bacteria can influence the host’s immune development and modulate its immunity, being important for disease prevention. Furthermore, less abundant taxa can be essential in shaping the gut microbial community, often being named “keystone species” due to their impact despite their lower abundance [64,65]. Low-abundance bacteria often act as drivers of compositional alteration after dietary changes and can be particularly important under nutrient-poor feeding conditions, as it will rely more on the capacities of microbes to aid digestion [64]. Due to their characteristics as keystone species, these low-abundant species can be important future focus points for establishing gut health biomarkers and helping diagnose malnutrition in aquaculture fish. In the present study, these taxa should be regarded as candidate biomarkers, as no independent validation or predictive framework was applied. Despite the exploratory nature of this study and the use of pooled samples, which does not allow complete exclusion of tank-level effects, the identified taxa provide a basis for future targeted validation studies.

## 5. Conclusions

In summary, this study provided valuable insights into the composition of meagre’s gut microbiota, laying the foundation for future research on the species’ gut health. Despite the high plant content in the experimental diets, meagre’s core microbiota appeared less sensitive to dietary changes than anticipated. However, it is important to emphasize the role of less-abundant taxa, which showed notable differences among fish with varying nutritional status. These taxa hold promise as targets for future research on gut health biomarkers in fish, and future studies should investigate both the mucosa and digesta microbiota to gain deeper insights into how these taxa influence their respective mucosal communities.

## Figures and Tables

**Table 1:** Ingredient composition and proximate analysis of the experimental diets.

	Experimental Diets		
	Control (CTRL)	Challenge (CD)	Extreme Challenge (ED)
**Ingredients (% dry weight basis)**			
Fish meal ^a^	55.1	15.0	5.0
CPSP ^b^	5.0	5.0	5.0
Wheat gluten ^c^	-	10.0	12.0
Corn gluten ^d^	-	11.6	14.3
Soybean meal ^e^	-	20.0	25.0
Rapeseed meal ^f^	-	7.5	11.0
Sunflower meal ^g^	-	5.0	7.5
Wheat meal ^h^	24.7	7.1	-
Fish oil	11.3	7.0	5.0
Soy oil	-	3.8	5.2
Rapeseed oil	-	3.8	5.2
Phosphate	-	0.4	1.1
Vitamin ^i^	1.0	1.0	1.0
Mineral ^j^	1.0	1.0	1.0
Choline	0.5	0.5	0.5
Binder	1.0	1.0	1.0
Taurine	0.3	0.3	0.3
**Proximate analysis (% dry matter basis)**			
Dry matter	92.3	95.0	93.0
Crude protein	49.0	46.4	47.3
Crude fat	18.1	18.1	17.9
Ash	10.0	7.1	7.0
Gross energy (kJ g^−1^ DM)	23.0	24.1	23.9

CP—crude protein; CF—crude fat. ^a^ Sorgal, S.A. Ovar, Portugal (CP—65.8%; CF—8.0%). ^b^ Soluble fish protein concentrate. Sopropèche G, France (CP: 77.0% DM; CL: 18.4% DM). ^c^ Sorgal, S.A. Ovar, Portugal (CP—83.0%; CF—2.3%). ^d^ Sorgal, S.A. Ovar, Portugal (CP—69.9%; CF—3.3%). ^e^ Sorgal, S.A. Ovar, Portugal (CP—51.3%; CF—1.1%). ^f^ Sorgal, S.A. Ovar, Portugal (CP: 39.9% DM; CL: 2.9% DM). ^g^ Sorgal, S.A. Ovar, Portugal (CP: 33.0% DM; CL 1.7%DM). ^h^ Sorgal, S.A. Ovar, Portugal (CP—12.2%; CF—1.2%). ^i^ Vitamins (mg kg^−1^ diet): retinol, 18,000 (IU kg^−1^ diet); cholecalciferol, 2000 (IU kg^−1^ diet); alpha tocopherol, 35; menadion sodium bisulphate, 10; thiamin, 15; riboflavin, 25; Ca pantothenate, 50; nicotinic acid, 200; pyridoxine, 5; folic acid, 10; cyanocobalamin, 0.02; biotin, 1.5; ascorbyl monophosphate, 50; inositol, 400. ^j^ Minerals (mg kg^−1^ diet): cobalt sulphate, 1.91; copper sulphate, 19.6; iron sulphate, 200; sodium fluoride, 2.21; potassium iodide, 0.78; magnesium oxide, 830; manganese oxide, 26; sodium selenite, 0.66; zinc oxide, 37.5; potassium chloride, 1.15 (g kg^−1^ diet); sodium chloride, 0.44 (g kg^−1^ diet).

**Table 2:** Ecological parameters of the intestinal microbiota of meagre (*Argyrosomus regius*) fed the experimental diets.

	CTRL	CD	ED
Richness ^1^	74 ± 70	84 ± 51	103 ± 78
Diversity ^2^	1.41 ± 1.18	1.04 ± 0.97	1.77 ± 1.42
Evenness ^3^	0.43 ± 0.38	0.31 ± 0.31	0.49 ± 0.36

Values presented as means ± standard deviation (±SD). n = 18 pooled samples per treatment (six pools per tank; three tanks per treatment). ^1^ Chao1 species richness: S_Chao1_ = S_obs_ + n_1_^2^/2n_2_, where S_obs_ is the observed number of species and n_1_ singletons, and n_2_ doubletons; ^2^ Shannon’s diversity index: H’ = −Σ(P_i_(lnP_i_)), whereas P_i_ is the number of individuals of the *i*th species; ^3^ Simpson’s Evenness Index: E = (1/ΣPi^2^)/S, where S is the number of species.

## Data Availability

The original contributions presented in this study are included in the article/Supplementary Material. Further inquiries can be directed to the corresponding author.

## Supplementary Materials

The following supporting information can be downloaded at: https://www.mdpi.com/article/10.3390/ani16030407/s1, Table S1: Zootechnical performance of meagre (*Argyrosomus regius*) fed the experimental diets; Table S2: MiSeq general sequencing data obtained from samples of Meagre juveniles fed the experimental diets CTRL, CD and ED; Table S3: Abundance of valid ASVs found in samples of Meagre juveniles fed the experimental diets CTRL, CD and ED; Table S4: Abundance of filtered ASVs found in samples of Meagre juveniles fed the experimental diets CTRL, CD and ED; Table S5: Abundance of highly filtered ASVs found in samples of Meagre juveniles fed the experimental diets CTRL, CD and ED; Table S6: Relative abundance of ASVs per taxa found in Meagre juveniles fed the experimental diets CTRL, CD and ED.

## Abbreviations

The following abbreviations are used in this manuscript:

NGS	Next-generation sequencing
ASV	Amplicon sequence variants
DGGE	Denaturing Gradient Gel Electrophoresis
FM	Fish meal
FO	Fish oil
