# Supplementary material for: Gut Microbiota Response in Meagre (Argyrosomus regius) Subjected to a Plant-Based Nutritional Challenge
Source: Animals (Basel). 2026 Jan 28;16(3):407. doi: 10.3390/ani16030407 (PMC12896953; doi:10.3390/ani16030407)
Supplement: Supplementary file 1 [file animals-16-00407-s001.zip › SupplementaryMaterialRevised_Microbiota1.pdf]

Table S1. Zootechnical performance of meagre (*Argyrosomus regius*) fed the experimental diets (average initial body weight of 4.6 ± 0.4 g)

	CTRL	CD	ED	p-value
**Final weight (g)**	48.7 ± 1.0^{c}	30.0 ± 0.6^{b}	19.3 ± 1.0^{a}	<0.001
**Weight gain (g)**	44.2 ± 1.0^{c}	25.4 ± 0.6^{b}	14.9 ± 1.1^{a}	<0.001
**Feed Intake (g kg ABW^{-1} day^{-1})**	39.1 ± 1.1^{a}	38.7 ± 1.2^{a}	46.98 ± 1.7^{b}	<0.001
**Feed efficiency**	0.85 ± 0.03^{c}	0.76 ± 0.03^{b}	0.53 ± 0.04^{a}	<0.001
**Daily growth index**	3.99 ± 0.05^{c}	2.90 ± 0.04^{b}	2.07 ± 0.11^{a}	<0.001

Mean values and standard deviation (±SD). Different letters in the same row stand for statistical differences between diets ($p < 0.05$). Daily growth index: ((final body weight$^{1/3}$ − initial body weight$^{1/3}$)/time in days) × 100. Feed efficiency: wet weight gain/dry feed intake. Diets: control (CTRL), challenge (CD), extreme challenge (ED).
